# Ethnic differential item functioning in the assessment of quality of life in cancer patients

**DOI:** 10.1186/1477-7525-3-60

**Published:** 2005-10-07

**Authors:** Ian S Pagano, Carolyn C Gotay

**Affiliations:** 1 Cancer Research Center of Hawaii, Honolulu, HI 96813, USA

**Keywords:** differential item functioning, ethnicity, item response theory, quality of life

## Abstract

**Background:**

Past research has shown that Filipino cancer patients report lower levels of quality of life (QoL) than other ethnic groups. One possible explanation for this is that Filipinos do not define QoL in the same manner as others, resulting in bias in their assessments. Hence, Filipinos would not necessarily have lower QoL.

**Methods:**

Item response theory methods were used to assess differential item functioning (DIF) in the quality of life (measured by the EORTC QLQ-C30) of cancer patients across four ethnic groups (Caucasian, Filipino, Hawaiian, and Japanese). The sample consisted of 359 cancer patients.

**Results:**

Results showed the presence of DIF on several items, indicating ethnic differences in the assessment of quality of life. Relative to the Caucasian and Japanese groups, items related to physical functioning, cognitive functioning, social functioning, nausea and vomiting, and financial difficulties exhibited DIF for Filipinos. On these items Filipinos exhibited either higher or lower QoL scores, even though their overall QoL was the same.

**Conclusion:**

This evidence may explain why Filipinos have previously been found to have lower overall QoL. Although Filipinos score lower on QoL than other groups, this may not reflect lower QoL, but rather differences in how QoL is defined. The presence of DIF did not appear, however, to alter the psychometric properties of the QLQ-C30.

## Background

In recent years medical researchers have shown increasing interest in the physical, psychological, and social health of individuals suffering from disease and treatment-related toxicity [1-3]. These broad characteristics are generally grouped under the inclusive heading *quality of life *(QoL), and offer a contrast to the more traditional biomedical markers, such as survival time or disease remission. A general definition of QoL is patients' perspectives on their ability to live useful and fulfilling lives, as influenced by, but not completely dependent on disease and treatment [1]. As an instrument of measurement in the clinical setting, QoL is defined functionally by patients' own perceptions of their performance in physical, occupational, psychological, social, financial, and somatic (i.e., physical symptomatology) areas [4,5].

The QoL construct is an important one with respect to measuring disease progress and treatment effectiveness. Because treatments for health conditions often have both short and long-term sequelae, including pain, fatigue, and depression, biomedical markers often fail to give a complete picture of patient status. Even when a person is disease free, the individual may still suffer from debilitating physical and mental anguish. Similarly, a shorter survival prognosis may be less onerous if the time remaining can be lived with enjoyment. Numerous areas of medical research, including heart disease, diabetes, arthritis, pharmacology, mental disorders, aging, and trauma are now examining QoL.

Cancer research is one area in particular that has shown an increased amount of QoL studies. The assessment of sequelae resulting from cancer therapies is an essential part of the cancer treatment process. One reason for this is that cancer treatments often involve therapies such as chemotherapy and radiation, which have high toxicities. Studies have shown that patients often experience fatigue [6], pain, sleep difficulties [7], depression [8,9], and sexual dysfunction [10], both during and after cancer treatment. Clearly, the impact that cancer has on QoL, both from the disease itself as well as its treatment, is significant. As a result, many prominent and important groups have expressed the need for QoL measures. Among these are international cancer institutes and societies [11], clinical trial groups [12], regulatory agencies [13], and the pharmaceutical industry [14].

While few would deny the importance of QoL outcomes in addition to biomedical markers, QoL assessment is more challenging. Because QoL is subjective [15], its assessment almost invariably requires patient ratings for measurement, and the outcome measures of interest are certain to contain measurement error. This measurement error can lead to invalid assessments of QoL. Therefore, questionnaire development and continuing validation are essential for the evaluation of QoL.

Although research suggests that that reliable data for QoL exists, potential differences between ethnic groups have not been fully examined. These can affect people's responses and thus estimates of reliability and validity [16-19]. Assessment of QoL is primarily reflective of Western concepts of illness, and it is not known whether these concepts are consistent in other communities (e.g., African, Asian, or Pacific Islander). In the Western view, illness is perceived primarily as an external disturbance that prevents an otherwise self-determined life course. However, many cultures do not share this perspective. Fatalism, karma, and cultural predeterminism play vital roles in their belief systems and illness is considered to be an integral part of one's life journey [5]. These contrasting views of illness are likely to create different perceptions of QoL in relation to illness. Therefore, cross-cultural studies are needed to provide a more complete picture of the multifaceted QoL construct [20].

In one multiethnic study, Gotay et al. [21] found that Filipinos have lower QoL, as measure by the QLQ-C30, when compared to Japanese and Caucasians. This finding persisted even after the effects of cancer stage, comorbidity, treatment, age, education, marital status, and place of birth had been controlled. Ethnicity explained as much as 8% of the variance in QoL after controlling for these factors. There are several possible causes for this ethnic difference. One is that there is a characteristic of either Filipino culture or Filipino genetics that results in lower QoL. If so, it is important to establish what it is, so that improvements could hopefully be made. However, another potential cause has to do with how QoL is measured. It is possible that the definition of QoL is not the same for Filipinos as it is for the Caucasian and Japanese groups, and that measured ethnic differences are not reflective of true differences in QoL.

An important but often overlooked aspect of questionnaire validation is the evaluation of *differential item functioning *(DIF, formerly called item bias). DIF occurs when one group of individuals responds differently from another group on a given questionnaire item, even though both groups are equivalent on the underlying construct that is assessed. For example, assume that the underlying construct assessed by the Scholastic Aptitude Test (SAT) is equivalent across males and females, but on the instrument there exists a question (an item) that is answered correctly more often by women than by men. This item is exhibiting DIF with respect to gender: It is biased against males in favor of females.

Confusion often exists regarding the use of the term DIF as opposed to item bias. When all of the items on a questionnaire are measuring the same global construct, and two groups have the same overall *ability *(i.e., the same average level of the construct of interest), DIF and item bias are equivalent. However, if the groups being compared are not equal in the underlying construct being measured, items exhibiting DIF are not necessarily biased and biased items will not necessarily show DIF. For example, suppose that women are of higher ability than men with respect to the construct assessed by the SAT. If no attempt were made to statistically control for overall ability, all items would be expected to show DIF with women outperforming men. Therefore, items reflecting DIF with women showing greater ability would not be biased, and items showing men and women as equal (no DIF) would be biased in favor of men. For this reason, it is important to statistically control (see below) for overall ability before assessing DIF.

When DIF is present, a questionnaire's validity becomes questionable and its generalizability is reduced. It may be more valid for one group of individuals than another. With SAT scores, this would imply that two people of equal ability, but from different groups, would not receive the same score. Although traditionally most research in DIF has been conducted involving achievement data such as SAT scores (the Educational Testing Service, ETS, began examining and removing items based on DIF with respect to ethnicity in 1986), the same principles apply to medical data involving QoL. When assessing QoL, DIF implies that two people with the same underlying QoL, who are from different groups, will not receive equivalent scores.

Research has shown that ethnicity does lead to potential DIF problems in quality of life [22,23], and on cognitive screening tests [24]. In comparing Danish and U.S. samples, Bjorner et al. [23] found DIF on 12 out of 35 items on the SF-36 Health Survey. Johnson et al. [22] looked at three QoL-related measures, the Sickness Impact Profile, the Ferrans and Powers' Quality of Life Index, and the Adult Self-Image Scales, and found that African-Americans had lower functional and affective scores when compared to Caucasians. Teresi et al. [24] found that an item related to remembering was less likely to be endorsed by Latinos than by Caucasians or African-Americans.

If ethnic DIF is present in quality of life, it might imply that the existence of ethnic differences in QoL is a result of biased items, and not real differences in the population. This would have implications for the assessment of QoL when working with multi-ethnic samples. In addition, reliability and validity measures obtained from previous studies may be suspect.

The primary goal of the present study was to establish whether or not DIF existed across ethnic groups for any of the items on the QLQ-C30. If DIF was found to exist, a second goal was to establish how this might have affected assessments of the instrument's psychometric properties. In this study conducted with patients living in Hawai'i, it was hypothesized that there would be a tendency for items to be biased with respect to Filipinos when compared to Japanese and Caucasians. That is, on certain items Filipinos would respond with either higher or lower QoL scores, even though their overall QoL was the same. If supported, this would suggest that ethnic differences in QoL, as measured by the QLQ-C30, do not necessarily reflect real QoL differences, but rather differences in how ethnic groups define QoL.

## Methods

### Participants

The study sample consisted of 366 cancer patients, 56% of the 646 eligible patients who were invited to participate. The most frequent reasons for nonparticipation were not feeling well enough to take part and being "not interested." Of the participants, 56% were women, 70% were married, 40% had a high school education or less, and the mean age was 62 years (standard deviation = 12.7). Ethnic breakdowns were 129 (35%) Japanese, 124 (34%) Caucasian, 61 (17%) Filipino, 42 (11%) Hawaiian, and 10 (3%) unknown. The most common cancer site was the breast (34%), followed by the prostate (28%). Most patients had received surgical treatment (83%), with several receiving radiation (42%), chemotherapy (20%), or hormonal treatment (25%).

Participants were identified through registrations on the Hawai'i Tumor Registry (HTR), a member of the National Cancer Institute-supported Surveillance, Epidemiology, and End Results Registry, which maintains records for all cancers diagnosed in the state. Eligibility criteria were histologic confirmation of any kind of cancer diagnosed between four and six months previously; ability to understand English; permission of primary physician; Oahu residency; Caucasian, Filipino, Hawaiian, or Japanese ethnic origin; and 18 years of age or older. Participation was not limited by stage or site of disease, but not all cancer sites were represented (e.g., no patients had colorectal, head and neck, lung, or ovarian cancer).

### Procedures

Permission to approach patients for this study was obtained from the attending physician before patients were contacted. Patients received a letter introducing the study's intent, followed by a telephone call to set appointments. Data were collected by interviews, most often at the patient's home. (In some circumstances the patients preferred to be interviewed at the Cancer Research Center of Hawaii.) Data were also abstracted from the chart for age, ethnicity, sex, marital status, and site and stage of cancer. Asking patients to indicate the ethnicity of their four grandparents verified patient ethnicity. Standard state criteria were used. Three of four grandparents from the same ethnic group defined a patient's ethnicity, except for Hawaiians, for whom any grandparent being Hawaiian superseded other ethnic classifications and resulted in the patient as being defined as Hawaiian. Interviews were conducted by one of four female research associates, all of whom had completed graduate work in social sciences as well as extensive training in interviewing cancer patients. Interviews took an average of one hour.

### Measures

During the participant interviews the questions from the EORTC QLQ-C30 version 1.0 were self-administered [3]. The questionnaire consists of 30 items each written to assess aspects of QoL (see Appendix [additional file 1]). The items are grouped into five functional scales (physical, role, cognitive, emotional, and social), three symptom scales (pain, fatigue, and nausea / vomiting), one global health status scale, five symptom items (dyspnea, insomnia, appetite loss, constipation, and diarrhea) and one financial difficulties item. Responses are either dichotomous (yes or no) for the physical and role scales, or Likert-type for the others.

Additionally, questions assessing Karnofsky Performance Status (KPS) [25] and receipt of chemotherapy were asked. This information was obtained to assess the correlations with the QLQ-C30 as a validity measure. The KPS has been frequently employed in clinical trials and QoL research [26], and assesses a person's ability to perform normal activities. The scale ranges from zero (deceased) to 100 (no impairment). The KPS is generally rated by an observer, and in this study, the interviewers were trained to provide a KPS score for each patient. Chemotherapy is a treatment known to have a significant impact on QoL and was recorded as either receipt of chemotherapy (coded one) or no chemotherapy received (coded zero).

### Data Analysis

Several methods have been developed for measuring DIF [27], of which two major categories exist: classical and modern. Modern psychometrics is mostly defined by item response theory (IRT), and this technique provides important advantages over the classical methods (commonly referred to as classical test theory, or simply CTT) [28-32]. When the assumption of unidimensionality (i.e., a single latent trait is influencing the items) is met, *invariance *and *information *are the two primary advantages of IRT over CTT modeling. Under invariance, item difficulties (see below) are independent of the sample (i.e., independent of overall ability) and person abilities are independent of the items (i.e., independent of the particular items responded to). IRT models have the invariance property because both item and person characteristics are estimated simultaneously within the model. Information refers to the item information function (IIF) that is computed for each item through IRT modeling. The IIF indicates how well an item discriminates between persons at varying ability levels.

Because of these advantages, an IRT approach has been adopted for the present analyses. Specifically, a one-parameter (Rasch) model using the marginal maximum likelihood procedure has been employed. The one-parameter model was chosen over the two-parameter model because it provides more invariant estimates of the item difficulties and requires fewer cases [33]. In Rasch modeling the slope (discrimination) parameter is set to 1.0 for all of the items. Detailed explanations of the one- and two-parameter IRT models, and maximum likelihood estimation can be found in the literature [28,30-34].

Even though Rasch modeling requires fewer cases than more complex IRT models, the number of individuals available in our sample was still less than desired. In IRT modeling, the general standard of reliability is to have 99% confidence that the parameter estimates are within one half logit of the stable value. To meet this standard using Rasch models, a minimum sample of 108 is recommended [35]. The Japanese and Caucasian groups are above this minimum, but the Hawaiian and Filipino groups are not. However, even with the smallest group, Hawaiian, the confidence levels obtained are still within an interpretable range. The Hawaiian and Filipino groups are both large enough to have 99% confidence that the parameter estimates are within one logit of the stable value [35]. Therefore, while extra caution is needed when interpreting the analyses in the Hawaiian and Filipino groups, potentially meaningful results can still be obtained.

Because the purpose of this study was to assess DIF resulting from ethnic differences, the data were grouped into four ethnic categories: Caucasian, Japanese, Filipino, and Hawaiian. IRT models were calculated for each ethnic group using the PARSCALE software application. The entire QLQ-C30 was used for the IRT models and not subcategories based on functional and symptom scales. Although Cella et al. [1] recommended using subcategories, the present research was interested in DIF with respect to the higher-order QoL construct and not the specific subcategories. The presence of this single higher-order construct is supported by research conducted by Gotay et al. [36] that employed confirmatory factor analysis.

Using the above IRT model within each ethnic group, an ability score was obtained for each participant (recall that ability refers to the level of the construct assessed, in this case QoL) and a *difficulty *score was obtained for each item. The term difficulty is borrowed from IRT models used to assess item difficulties for educational testing. More difficult items are ones that are less likely to be answered correctly. When assessing QoL there is clearly no right or wrong answer. However, even though difficulty may seem an inappropriate term when assessing QoL, it still in fact conveys the same type of information.

For an educational testing measure, more difficult items are those that require a higher ability in order to be answered in the direction of higher ability (i.e., correctly). For a quality of life measure, more difficult items are those that require a higher QoL in order to be answered in the direction of higher QoL. For example, question 2 from the QLQ-C30 asks, "Do you have any trouble taking a long walk?" and question 3 asks, "Do you have any trouble taking a short walk outside of the house?" It is reasonable to suggest that a person would need a higher QoL to take a long walk than to take a short one. Therefore, it is more difficult to provide a "no" response to question 2 than it is for question 3.

After obtaining the item difficulties, group differences that were consistent across all items (and were assumed to be reflective of true differences in the population) were statistically controlled by standardizing the item difficulties within each ethnic group [32]. This ensured that the means and standard deviations were equivalent across groups, and that the model parameters were on a common scale. Hence, items found to exhibit DIF reflected the presence of bias, and not actual group differences. Using the item difficulties, intraclass correlations (note that because the item difficulties were standardized, the intraclass correlations are equivalent to Pearson correlations) were calculated between each ethnic group. Two groups are considered to be of the same type (i.e., having equivalent rank order of the item difficulties) when the correlation between them is greater than 0.98 [37].

Next, the analysis of differential item functioning was made using the PARSCALE software. Because of the number of items that were analyzed (*n *= 30), the α-level was set to .0017 (using the Bonferroni adjustment, 0.05 / 30). Each item difficulty was compared across all four groups and the items that showed statistically significant differences exhibited DIF, which implied item bias.

Finally, an examination of the reliability and validity (validity was assessed using concurrent measures) of the QLQ-C30 was made with both the original unadjusted QLQ-C30 scores and with DIF-adjusted scores. Two methods were used for calculating the adjusted scores. The first involved simply removing the items shown to exhibit DIF. For the second, the effect of ethnicity was partialed out of the DIF items using standard regression techniques for computing a partial correlation. Reliability was assessed with coefficient α. Calculating the correlation between overall QLQ-C30 score and two measures known to be related to QoL, receipt of chemotherapy (yes or no) and the Karnofsky Performance Status Scale [38], assessed validity. When comparing the results from the unadjusted and adjusted measures, a small change in reliability and validity would be consistent with a hypothesis that the QLQ-C30 maintains the same psychometric properties even in the presence of DIF.

## Results

Prior to conducting analyses, seven of the 366 patients were removed from the sample because their ethnicity was unknown. Missing values for items on the QLQ-C30 were replaced by the mean values for the respective ethnic groups. For example, if an individual who was Japanese left a question blank, the value was replaced by the mean of the given question for the Japanese group (rounded to the nearest integer value). Out of 10,770 possible values (359 patients multiplied by 30 items on the QLQ-C30), there were only 11 (0.1%) missing values that needed to be imputed in this way. Patient characteristics are shown in Table 1.

**Table 1:** Means (M) and standard deviations (s) of demographic and cancer measures

	**Ethnicity**									
**Measure**	**Caucasian** n = 121		**Japanese** n = 133		**Filipino** n = 61		**Hawaiian** n = 51		**Total** n = 366	
	M	s	M	s	M	s	M	s	M	s
Age*	63.4	11.8	63.7	11.9	58.6	13.4	57.3	14.6	61.9	12.8
Female (%)	51.2	50.2	58.6	49.4	50.8	50.4	64.7	48.3	55.7	49.7
Education (years)*	14.2	3.0	12.9	2.9	11.9	4.0	12.1	2.6	13.0	3.2
Married (%)	63.0	48.5	73.5	44.3	76.3	42.9	59.2	49.7	68.5	46.5
Cancer Site										
Breast (%)*	29.8	45.9	44.4	49.9	19.7	40.1	37.3	48.8	34.4	47.6
Prostate (%)	32.2	46.9	27.1	44.6	31.1	46.7	11.8	32.5	27.3	44.6
Advanced Stage (%)	13.2	34.0	9.0	28.8	25.0	43.7	7.8	27.2	12.9	33.5
Performance Status	87.4	11.0	87.7	10.0	84.3	10.4	85.1	11.4	86.7	10.6
Comorbidity (%)	58.7	49.4	62.4	48.6	55.7	50.1	62.7	48.8	60.1	49.0
Chemo (%)	7.5	26.4	10.5	30.8	15.3	36.3	12.5	33.4	10.6	30.8
Hormone (%)	19.1	39.5	30.6	46.3	16.1	37.1	25.0	43.8	23.7	42.6
Radiation (%)	37.8	48.7	43.7	49.8	27.6	45.1	27.1	44.9	36.8	48.3
Surgery (%)	83.5	37.3	80.3	39.9	77.0	42.4	94.1	23.8	82.7	37.8

Notes. Advanced Stage was defined as regional or distant disease. Performance Status is the Karnofsky Performance Status, which ranges from 0 to 100 (where 0 = deceased and 100 = no sign of disease). Comorbidity indicates the presence of a comorbid disease. Chemo, Hormone, Radiation, and Surgery indicate receipt of the respective treatment. Statistically significant (p < .01) differences across ethnic groups are indicated with an asterisk (*).

The intraclass correlations between group difficulties of the items are shown in Table 2. An examination of the values indicates that none of the ethnic groups is exactly the same type (using a cutoff intraclass correlation of 0.98 [37]), although the Caucasian and Japanese groups are the closest (0.945). The two groups most different from one another are the Hawaiians and Filipinos (0.792). These intraclass correlations give an indication of how many individual items will show significant DIF. Because the Hawaiians and Filipinos have the lowest intraclass correlation, these groups can be expected to have the largest number of items with significant DIF. The groups with the highest intraclass correlation, Caucasians and Japanese, can be expected to have the least.

**Table 2:** Item Difficulty Intraclass Correlations by Ethnic Group

	**Caucasian**	**Japanese**	**Filipino**	**Hawaiian**
**Caucasian**				
**Japanese**	0.945			
**Filipino**	0.896	0.863		
**Hawaiian**	0.871	0.939	0.792	

Note. Because the item difficulties were standardized (mean = 0, standard deviation = 1), the intraclass correlations are equivalent to the Pearson correlations.

Of the 30 items examined, 12 (40%) showed significant DIF (see Table 3 for the IRT difficulty parameters and significance tests across all of the ethnic groups). When compared to Caucasians, five items existed that were significantly biased against Filipinos, and three that were significantly biased in favor. When compared to Japanese, three items existed that were significantly biased against Filipinos, and five were significantly biased in favor. This supports the stated hypothesis that biased items would exist for Filipinos when compared to Caucasian and Japanese groups. Further, when Filipinos were compared to Hawaiians, there were five significantly biased items. The items that appear consistently biased against Filipinos, independent of the comparison group, are ones representing nausea and vomiting (items14 and 15) and financial difficulties (item 28). Items that appear consistently biased for Filipinos are one representing physical functioning (items 1, 2, and 29) and cognitive functioning (item 25, remembering).

**Table 3:** IRT difficulty parameters for the QLQ-C30 items

**Standardized Difficulty**							
Item	Content	**Caucasian**	**Japanese**	**Filipino**	**Hawaiian**	χ^2^(3)	***p***
01	Physical	*1.09*	**1.64**	*1.11*	*0.74*	27.7	*
02	Physical	0.72	**0.83**	*0.19*	**1.11**	15.2	*
03	Physical	-1.51	-1.29	-2.35	-0.61	11.1	.01
04	Physical	-1.12	-0.78	-1.24	-0.88	2.3	.51
05	Physical	-2.15	-2.39	-2.97	-2.29	2.5	.47
06	Role	0.81	0.51	0.51	0.61	7.3	.06
07	Role	*-1.79*	**-0.80**	**-0.73**	**-0.38**	22.2	*
08	Dyspnea	-0.15	-0.01	-0.06	0.17	4.2	.24
09	Pain	0.90	0.89	0.67	0.68	5.3	.15
10	Fatigue	**1.50**	*0.94*	1.25	1.02	28.8	*
11	Insomnia	1.05	0.68	0.93	1.02	11.0	.01
12	Fatigue	0.23	0.35	0.51	0.43	5.2	.15
13	Appetite Loss	-0.11	-0.39	0.00	-0.34	5.2	.16
14	Nausea	*-0.96*	*-1.25*	**-0.30**	-0.61	19.4	*
15	Nausea	*-2.23*	*-2.89*	**-1.35**	*-3.43*	19.5	*
16	Constipation	*-0.34*	0.01	**0.16**	**0.16**	27.2	*
17	Diarrhea	-0.44	-0.55	-0.73	-1.04	12.1	.01
18	Fatigue	1.26	1.20	1.08	1.41	1.3	.72
19	Pain	-0.35	-0.14	-0.13	0.11	9.0	.03
20	Cognitive	-0.42	-0.35	-0.42	-0.24	1.0	.80
21	Emotional	0.44	0.18	0.42	0.01	11.1	.01
22	Emotional	**1.18**	0.91	**1.27**	*0.56*	21.7	*
23	Emotional	0.49	0.51	0.58	0.72	2.5	.47
24	Emotional	0.47	0.30	0.54	-0.03	11.0	.01
25	Cognitive	**0.54**	**0.89**	*0.13*	**0.92**	30.2	*
26	Social	0.07	0.23	0.21	-0.24	4.7	.19
27	Social	**0.71**	**0.57**	*0.28*	*0.23*	15.2	*
28	Financial	*0.26*	*0.30*	**1.12**	*0.49*	47.0	*
29	Physical	**0.08**	**0.14**	*-0.33*	-0.01	15.1	*
30	Overall	-0.22	-0.22	-0.37	-0.31	2.0	.56

Notes. Standardized difficulty parameters are given for each ethnic group. For a given item, values shown in **bold **are significantly greater than values shown in italics. Values shown in plain type do not differ significantly from other values. The χ^2^(3) column provides the chi-square value (with 3 degrees of freedom) for the omnibus test for differences in difficulty across all four ethnic groups. The p column is the p-value for the chi-square test. An asterisk (*) indicates that the p-value is less the alpha-value, which was set to 0.0017 (0.05 / 30). Because Rasch modeling was used, all items have equal slope parameters, set to 1.0.

Some other findings related to item bias were also noteworthy. For one emotional functioning item (item 22, worry), Hawaiians show significantly less difficulty. For Caucasians, a role functioning question (item7, work at job) and the constipation question (item16) were significantly less difficult for them. For a social functioning item (item 27, social activities), Caucasians and Japanese had greater difficultly than Hawaiians and Filipinos.

Finally, an assessment of the reliability and validity of the QLQ-C30, both with the unadjusted overall QoL scores and the DIF-adjusted scores, were made. Coefficient α was calculated for the unadjusted scale, the scale adjusted by deleting DIF items, and the scale adjusted by partialling ethnicity from DIF items. The values were .94, .92, and .93, respectively.

The correlations between unadjusted overall QoL scores with receipt of chemotherapy and Karnofsky Performance Status were *r *= -.14 (*p *= .01) and *r *= .59 (*p *< .0001), respectively. After the removal of the QLQ-C30 items shown to exhibit DIF, the absolute values of the correlations dropped slightly to *r *= -.12 (*p *= .02) and *r *= .56 (*p *< .0001). Using the item partialling adjustment, correlations of *r *= -.13 (*p *= .02) and *r *= .58 (*p *< .0001) were obtained. The low correlation between chemotherapy and QoL may be related to persons with high cancer stage being less likely to receive chemotherapy.

Note that adjustment for DIF (both the deletion and partialling methods) resulted in a reduction in the level of ethnic differences for overall QoL score. This in turn caused the variance in QoL score to also be reduced, which led to the lower α coefficients and lower correlations. The reduction was too small, however, for it to be considered a result of anything other than a mathematical artifact, suggesting that DIF may not have an impact on the psychometric properties of the QLQ-C30.

## Discussion

### Overview

Differential item functioning was found in several of the items contained in the EORTC QLQ-C30, implying that not all of the items assess quality of life equally across ethnicity. This supports theories and research suggesting that ethnicity influences perceptions of health and sickness [16-19], and indicates that caution is necessary when comparing scores between ethnic groups. Because responses to the questionnaire are dependent on a factor unrelated to QoL, namely ethnicity, persons who are equal on the underlying construct of QoL will not necessarily respond equally on the questionnaire. Should it be necessary to compare QoL scores between ethnic groups, a special consideration must be made for those items that exhibit DIF.

Several items existed that showed statistically significant bias with respect to Filipinos, supporting the main hypothesis of the study. The factors of physical functioning, nausea and vomiting, cognitive functioning, social functioning, and financial difficulties are potentially biased. This implies that Filipinos will indicate either higher or lower scores on these items when compared to the other ethnic groups, even when their global QoL is the same. Hence, the global QoL as measured by the QLQ-C30 will be biased with respect to Filipinos.

However, the presence of DIF did not appear to alter reliability and validity estimates. Neither removal nor adjustment of items shown to exhibit DIF had an appreciable impact on any of the estimated values. Although replication is clearly needed to support this and all of the findings presented here, this suggests that the deletion or modification of the items exhibiting DIF is not necessary, and may even be inappropriate. So, assuming the existence of DIF is reflective of real ethnic differences in how certain items are interpreted, what are its implications and how should it be addressed?

## Implications

Although detecting DIF is relatively straightforward, determining its cause and the best method to alleviate its presence is more difficult. If the reasons for DIF had been suspected to arise because of poorly written questions that are not interpreted consistently, then rewording or even removal of items is probably necessary. Even though the QLQ-C30 has been tested to ensure that the questions are worded properly [39], there still remains the possibility that ethnic groups not previously examined will have different interpretations. However, in the present sample, all individuals were English speaking, lived in Hawai'i, and had the opportunity to ask for clarification if they were confused. Therefore, the likelihood of poor wording being the primary cause of the DIF found here is reduced, and rewording or removal of items is probably unwarranted.

If poor wording or misinterpretation of items cannot explain the presence of DIF, then this suggests that the definition of QoL is not equivalent across ethnic groups. The questions exhibiting DIF provide relevant information for assessing how these definitions differ. Specifically, the DIF questions are not consistent measures of the QoL construct across all ethnic groups, providing little QoL-related information for one ethnic group but valuable information for another. Hence, not only is it important that these items be kept, but it is necessary that special attention be paid to them when comparing QoL across ethnic groups.

An examination of the items showing significant DIF between the Caucasians and the other ethnic groups shows that Caucasians are better able to work at a job or to do household jobs, and experience less constipation. Because overall QoL had been controlled, this finding may suggest that Caucasians value these factors more strongly than others in terms of global QoL. In order for Caucasians to achieve the same level of QoL held by others they may need better performance with their work and they may need greater freedom from constipation. If the Caucasians were equal with others on these items, their underlying QoL would likely be less.

The Hawaiians, when compared with others, showed significantly lower scores on the emotional functioning item related to worry. This may suggest that for Hawaiians, when compared to the other groups, freedom from worry is more important in relation to their global QoL. Similarly, the items that were biased against the Filipinos may reflect aspects of QoL that are not particularly relevant to the Filipino population. For example, there was significant bias in the items reflecting financial difficulties. Perhaps in the Filipino worldview financial success is less of a necessity, and when hardships of costly treatments are endured, the impact on QoL is less severe.

An important question to consider for future research is what cultural differences could explain the different interpretation of certain items. For example, why did the item related to working appear to have greater importance for Caucasians? Perhaps in Caucasian society work attributes are more highly prized than in other societies. This is consistent with the current lore regarding Caucasian and Asian cultures. When compared to Asians, Caucasians place greater value on independence and individual achievement [40]. Therefore, for Caucasians, not being able to work may be particularly detrimental to quality of life. If so, this may have significance beyond simply the assessment of quality of life in cancer patients. This may provide important evidence for culture-based theories comparing individualist versus collectivist societies.

## Recommendations

It must be emphasized that before any recommendations can be made for adjusting scores or modifying the QLQ-C30 based on DIF, the results found here must be replicated. This is an exploratory study, which we hope will lead to increased research in this area. While this sample from Hawai'i certainly contributes to the current lore, it is by no means representative enough to indicate, by itself, how corrections can be made for a questionnaire that is used in countries all over the world. Future research needs to expand the study of DIF to additional countries, cultures, and ethnic groups. Through continued research in this area, a clearer picture will emerge as to how the definition of QoL varies across ethnic groups.

Assuming that the findings presented here are replicated and that future research demonstrates which items are consistently biased and to what degree the bias exists, one approach to correct DIF might be to weight items based on ethnicity. Items that are biased against a particular group should be given less weight in the calculation of overall QoL for individuals in that group. By placing greater emphasis on the items that are most relevant for a particular group, most biases could be eliminated. The weighting scheme should be based on the level of bias that exists. For example, items severely biased against a certain group should be given little weight when calculating that group's overall QoL score. But items biased in favor should be given greater weight. From the present study, it is suggested that items related to work and constipation should probably be given greater emphasis when assessing Caucasians. Similarly, items related to financial difficulties and nausea should probably be given less weight for Filipinos, whereas items related to physical functioning should be given more.

Another approach that could be used is similar to the one of the methods employed here for removing bias from the DIF items. If an item is biased, the effect of ethnicity could be partialed out of it using standard regression procedures (controlling for the effect of ethnicity). This would make all ethnic groups equivalent on the particular item. However, this approach could prove cumbersome in practice because additional statistical procedures would be required, as compared to simply weighting items.

Finally, it is recommended that DIF not be seen solely as a problem that is to be eliminated. Items exhibiting DIF may reflect key differences in how QoL is defined across ethnic groups, and important information can be obtained from these items for understanding cultural differences. Therefore, it is not recommended that any of the biased items be removed, or even reworded. Rather, expanded cultural studies should be undertaken to further explain the multifaceted QoL construct.

## Limitations

The primary strength of the methods employed here is the ability to detect isolated items exhibiting DIF (i.e., item bias). However, a limitation with this approach is that it cannot detect item bias if the bias exists within most or all of the items. For example, if all of the items on the QLQ-C30 were biased against Filipinos, then this approach would have failed to detect the biases. Group differences would have appeared to reflect genuine differences in QoL, and not bias. However, because of the extensive research that has been conducted assessing the validity of the QLQ-C30, the risk of such widespread bias is reduced.

Another limitation arises when statistically controlling for group differences by standardizing the item difficulties within each group. In order for this controlling procedure to be effective, it is necessary for relatively few of the items to be biased. If too many items are biased, then it is not necessarily only quality of life that is being controlled, but also factors related to the bias. In this study, the issue is probably not serious because there were many items (60%) that did not show statistically significant bias. With the possible exception of the Hawaiian and Filipino comparisons, the QLQ-C30 was suited for assessing item bias with the methods employed here.

Finally, a two-parameter IRT methodology may provide a greater level of item information (recall that information refers to how well an item discriminates between persons at varying ability levels). Some of the items on the QLQ-C30 may provide more information than others in assessing QoL, and a two-parameter model would indicate the amount of information provided by each item. In the present study, the sample size was not large enough to obtain an optimal IRT solution when incorporating two-parameters, but future QoL research is planned in which a two-parameter IRT model will be used. This future research will also examine each of the QoL subscales in addition to overall QoL, allowing for a comparison between the two approaches.

## Conclusion

In conclusion, this research suggests that quality of life, as assessed by the EORTC QLQ-C30, is at least partially dependent on one's ethnic origin. The culture from which one belongs is an important determinant of how one defines QoL. This may explain why previous research has shown Filipinos to have a lower QoL than other ethnic groups [21]. Filipinos may be equal to other groups with respect to underlying QoL, but different in terms of the aspects that characterize their QoL. This study suggests that persons from different ethnic backgrounds do not define QoL in exactly the same manner, and research involving QoL that employs different ethnic groups cannot ignore this important issue.

## Authors' contributions

IP designed the study, carried out all of the analyses, and drafted the manuscript.

CG handled the data collection and editing of the manuscript.

## Supplementary Material

Additional File 1 Pagano and Gotay Appendix.doc. Appendix: The QLQ?C30 version 1.0 with Functional / Symptom Scales Indicated Click here for file
