# Supplementary material for: Ethnic differential item functioning in the assessment of quality of life in cancer patients
Source: Health Qual Life Outcomes. 2005 Oct 7;3:60. doi: 10.1186/1477-7525-3-60 (PMC1277838; doi:10.1186/1477-7525-3-60)
Supplement: Additional File 1 — Pagano and Gotay Appendix.doc. Appendix: The QLQ?C30 version 1.0 with Functional / Symptom Scales Indicated [file 1477-7525-3-60-S1.doc]

# **Appendix**

**The** **QLQ‑C30 version 1.0 with Functional / Symptom Scales Indicated**

|  | | | | **Scale** | |  | | **No** | **Yes** | |  |
| --- | --- | --- | --- | --- | --- | --- | --- | --- | --- | --- | --- |
| 1. Do you have any trouble doing strenuous activities, like carrying a heavy shopping bag or a suitcase? | | | | Physical | |  | | 1 | 2 | |  |
| 1. Do you have any trouble taking a **long** walk? | | | | Physical | |  | | 1 | 2 | |  |
| 1. Do you have any trouble take a **short** walk outside of the house? | | | | Physical | |  | | 1 | 2 | |  |
| 1. Do have to stay in bed or a chair for most of the day? | | | | Physical | |  | | 1 | 2 | |  |
| 1. Do you need help with eating, dressing, washing yourself or using the toilet? | | | | Physical | |  | | 1 | 2 | |  |
| 1. Are you limited in any way in doing either your work or doing household jobs? | | | | Role | |  | | 1 | 2 | |  |
| 1. Are you completely unable to work at a job or to do household jobs? | | | | Role | |  | | 1 | 2 | |  |
|  | | | |  | |  | |  |  | |  |
| **During the past week:** | | | | **Scale** | | **Not at all** | | **A little** | **Quite a bit** | | **Very much** |
| 1. Were you short of breath? | | | | Dyspnoea | | 1 | | 2 | 3 | | 4 |
| 1. Have you had pain? | | | | Pain | | 1 | | 2 | 3 | | 4 |
| 10. Did you need rest? | | | | Fatigue | | 1 | | 2 | 3 | | 4 |
| 11. Have you had trouble sleeping? | | | | Insomnia | | 1 | | 2 | 3 | | 4 |
| 12. Have you felt weak? | | | | Fatigue | | 1 | | 2 | 3 | | 4 |
| 13. Have you lacked appetite? | | | | Appetite Loss | | 1 | | 2 | 3 | | 4 |
| 14. Have you felt nauseated? | | | | Nausea and Vomiting | | 1 | | 2 | 3 | | 4 |
| 15. Have you vomited? | | | | Nausea and Vomiting | | 1 | | 2 | 3 | | 4 |
| **During the past week:** | | | | **Scale** | | **Not at all** | | **A little** | **Quite a bit** | | **Very much** |
| 16. Have you been constipated? | | | | Constipation | | 1 | | 2 | 3 | | 4 |
| 17. Have you had diarrhoea? | | | | Diarrhoea | | 1 | | 2 | 3 | | 4 |
| 18. Were you tired? | | | | Fatigue | | 1 | | 2 | 3 | | 4 |
| 19. Did pain interfere with you daily activities? | | | | Pain | | 1 | | 2 | 3 | | 4 |
| 20. Have you had difficulty in concentrating on things, like reading a newspaper or watching television? | | | | Cognitive | | 1 | | 2 | 3 | | 4 |
| 21. Did you feel tense? | | | | Emotional | | 1 | | 2 | 3 | | 4 |
| 22. Did you worry? | | | | Emotional | | 1 | | 2 | 3 | | 4 |
| 23. Did you feel irritable? | | | | Emotional | | 1 | | 2 | 3 | | 4 |
| 24. Did you feel depressed? | | | | Emotional | | 1 | | 2 | 3 | | 4 |
| 25. Have you had difficulty remembering things? | | | | Cognitive | | 1 | | 2 | 3 | | 4 |
| 26. Has your physical condition or medical treatment interfered with your **family** life? | | | | Social | | 1 | | 2 | 3 | | 4 |
| 27. Has your physical condition or medical treatment interfered with your **social** activities? | | | | Social | | 1 | | 2 | 3 | | 4 |
| 28. Has your physical condition or medical treatment caused you financial difficulties? | | | | Financial Difficulties | | 1 | | 2 | 3 | | 4 |
|  | | | | | | | | | | | |
|  | | | | | | | | | | | |
| **Global Health Status** | | | | | | | | | | | |
| 29. How would you rate your overall **physical condition** during the past week? | | | | | | | | | | | |
| 1  Very poor | 2 | 3 | 4 | | 5 | | 6 | | | 7  Excellent | |
|  | | | | | | | | | | | |
| 30. How would you rate your overall **quality of life** during the past week? | | | | | | | | | | | |
| 1  Very poor | 2 | 3 | 4 | | 5 | | 6 | | | 7  Excellent | |
